# 178. Endemic Carbapenem Resistance Driven By Clonal and Horizontal Spread of *bla*_IMP-4_ Across Diverse Enterobacterales: Jumping Genes, Promiscuous Plasmids and Killer Clones

**DOI:** 10.1093/ofid/ofab466.178

**Published:** 2021-12-04

**Authors:** Nenad Macesic, Luke Blakeway, Adam W Jenney, Anton Peleg

**Affiliations:** 1 Monash University / Alfred Hospital, Melbourne, Victoria, Australia; 2 Alfred Hospital / Monash University, Melbourne, Victoria, Australia; 3 Alfred Health, Melbourne, Victoria, Australia

## Abstract

**Background:**

Carbapenem-resistant Enterobacterales (CRE) have become endemic and cause significant morbidity and mortality globally. The metallo-beta-lactamase gene *bla*_IMP-4_ is a key CRE resistance determinant in Australia and Asia but its genomic context remains unknown. We aimed to determine the genomic epidemiology of *bla*_IMP-4_ in clinical and environmental isolates from 2008 – 2020 at our institution.

**Methods:**

We performed whole genome sequencing on 219 *bla*_IMP-4_-carrying isolates from 134 patients (219 short-read and 75 long-read). Multi-locus sequence types (MLSTs), resistance determinants and plasmid replicons were assessed. High-quality *de novo* hybrid assemblies were used to identify location of *bla*_IMP-4_ gene. We conducted phylogenetic analysis for key MLSTs and plasmids.

**Results:**

*Bla*_IMP-4_ was noted on a class I integron also harboring aminoglycoside, sulfamethoxazole, chloramphenicol and quaternary ammonium compound resistance genes. This integron was able to migrate over time to 10 bacterial species (42 STs) and 6 different plasmid types (Figure 1 and Figure 2). From 2008-2020, *bla*_IMP-4_ was present on IncC plasmids in *Serratia marcescens* and *Klebsiella pneumoniae*. We noted small outbreaks of *Pseudomonas aeruginosa* ST111 with chromosomal integration of *bla*_IMP-4_ from 2008-2018 (16 isolates) and *Enterobacter cloacae* complex ST114 with *bla*_IMP-4_ on IncFIB(K)/IncFIA(HI1) plasmids from 2011-2020 (19 isolates). From 2016-2020, there was an explosion of diverse IncHI2 plasmids carrying *bla*_IMP-4_. This was driven by clonal expansion of *E. cloacae* complex ST93/ST190 (79 isolates), with spillover of IncHI2 plasmids to *Klebsiella* spp (13 isolates), *Citrobacter* spp (2 isolates), *S. marcescens* (1 isolate)*, Escherichia coli* (4 isolates). In addition to *bla*_IMP-4_, these plasmids carried *mcr-9.1*, a colistin resistance gene, and resistance determinants to nearly all key classes of Gram-negative antimicrobials.

Figure 1. Bacterial species harboring blaIMP-4 2008-2020

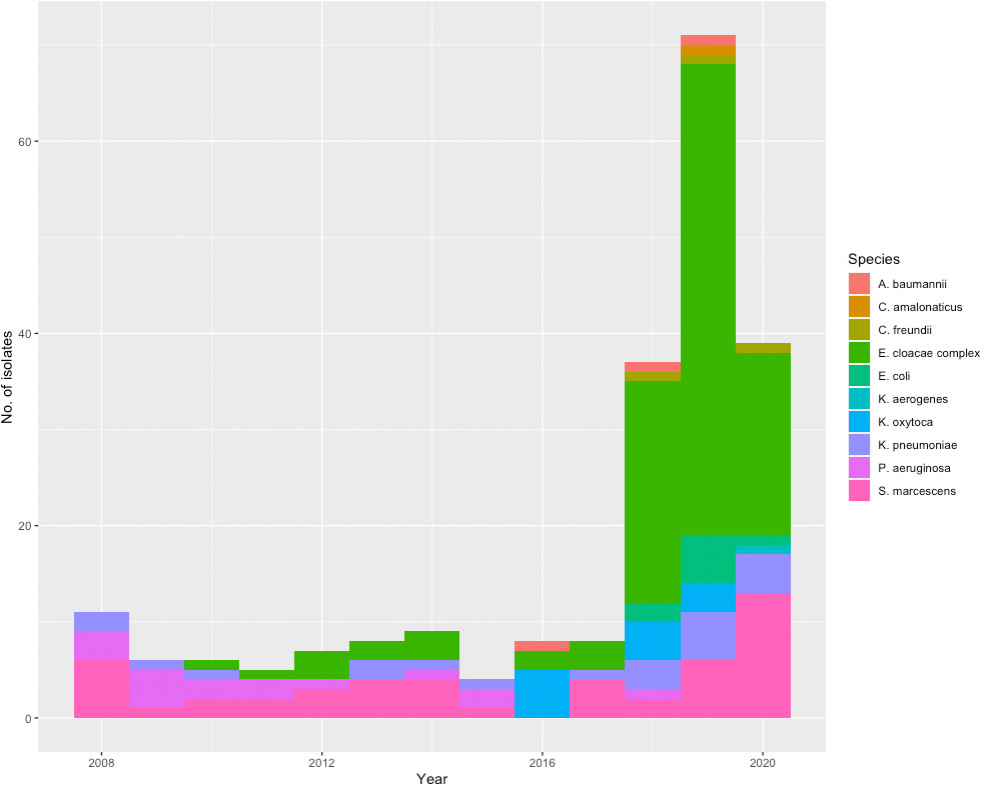

BlaIMP-4 was noted in diverse bacterial species over the study period. Serratia marcescens and Klebsiella pneumoniae were present throughout. Outbreaks of Enterobacter cloacae complex ST114, ST190 and ST93 and Pseudomonas aeruginosa ST111 were noted.

Figure 2. Diverse plasmids associated with blaIMP-4 carriage determined by de novo hybrid assembly

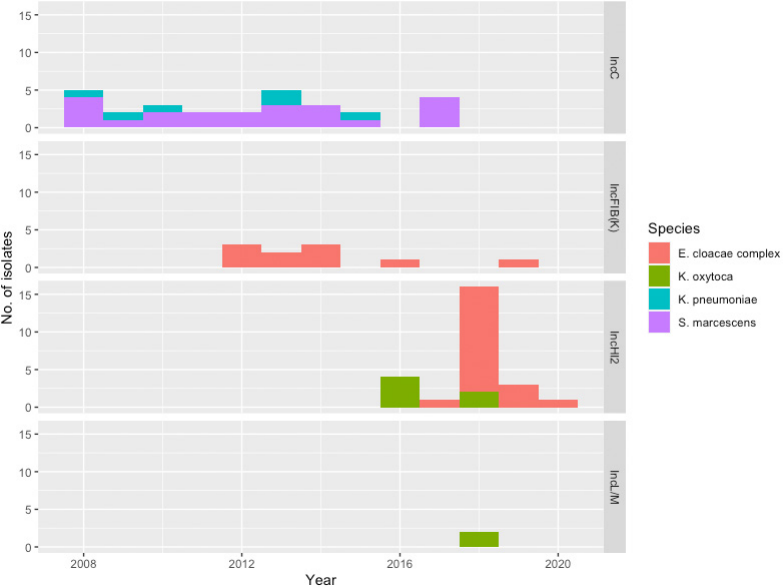

Presence of blaIMP-4 on diverse plasmids that varied through the study period was noted. Plasmids were charaterised by analysing de novo hybrid assembly data and co-location of blaIMP-4 and plasmid replicons on the same contigs.

**Conclusion:**

*Bla*_IMP-4_ spread on a class I integron was responsible for endemic carbapenem resistance at our institution. This mobile genetic element was able to persist due to both clonal spread and entry into diverse plasmids. Concerningly, we noted a large outbreak driven by IncHI2 plasmids harboring colistin resistance genes with spread to multiple bacterial species.

**Disclosures:**

**All Authors**: No reported disclosures

